# A randomized trial of a behavioral intervention to decrease hospital length of stay by decreasing bedrest

**DOI:** 10.1371/journal.pone.0226332

**Published:** 2020-01-10

**Authors:** Juliana Tolles, Gabriel Waterman, Charles E. Coffey, Rebecca Sandoval, Ross J. Fleischman, Mailee Hess, Laura Sarff, Roger J. Lewis, Brad Spellberg, on behalf of the BRAVE Study Group

**Affiliations:** 1 Department of Emergency Medicine, Harbor-University of California Los Angeles (UCLA) Medical Center, Torrance, California, United States of America; 2 David Geffen School of Medicine at UCLA, Los Angeles, California, United States of America; 3 CareMore Health, Pico Rivera, California, United States of America; 4 Los Angeles County + University of Southern California (USC) Medical Center, Los Angeles, California, United States of America; 5 Department of Medicine, Keck School of Medicine at USC Los Angeles, Los Angeles, California, United States of America; 6 Department of Medicine, Harbor-UCLA Medical Center, Torrance, California, United States of America; Ospedale San Raffaele, ITALY

## Abstract

**Background:**

Approximately half of hospitalized patients suffer functional decline due to spending the vast majority of their time in bed. Previous studies of early mobilization have demonstrated improvement in outcomes, but the interventions studied have been resource-intensive. We aimed to decrease the time hospital inpatients spend in bed through a pragmatic mobilization protocol.

**Methods:**

This prospective, non-blinded, controlled clinical trial assigned inpatients to the study wards per routine clinical care in an urban teaching hospital. All subjects on intervention wards were provided with a behavioral intervention, consisting of educational handouts, by the nursing staff. Half of the intervention wards were supplied with recliner chairs in which subjects could sit. The primary outcome was hospital length of stay. The secondary outcome was the ‘6-Clicks’ functional score.

**Results:**

During a 6-month study period, 6082 patient encounters were included. The median length of stay was 84 hours (IQR 44–175 hours) in the control group, 80 hours (IQR 44–155 hours) in the group who received the behavioral intervention alone, and 88 hours (IQR 44–185 hours) in the group that received both the behavioral intervention and the recliner chair. In the multivariate analysis, neither the behavioral intervention nor the provision of a recliner chair was associated with a significant decrease in length of stay or increase in functional status as measured by the ‘6-Clicks’ functional score.

**Conclusion:**

The program of educational handouts and provision of recliner chairs to discourage bed rest did not increase functional status or decrease length of stay for inpatients in a major urban academic center. Education and physical resources must be supplemented by other active interventions to reduce time spent in bed, functional decline, and length of stay.

**Trial registration:**

ClinicalTrials.gov, HS-16-00804.

## Introduction

Hospitalized patients spend the majority of their time in bed, even when they are capable of being out of bed. Up to 50% of inpatients suffer functional decline at least in part due to lying in bed after admission, and even those who are not placed on bed rest by their physicians spend an average of 20 out of 24 hours in bed [1–5]. The loss of functional ability and exercise tolerance happens remarkably fast, as early as hospital day 2 (24–48 hours) [1, 2, 6–8]. Functional decline often results in a prolonged hospital stay and the need for additional rehabilitation resources both during and after the hospitalization to return a patient to their baseline functional status [1–3, 6]. Functional decline has also been associated with increased mortality [1–4].

We sought to flip the culture of hospital-based care such that the bed was viewed as a place for patients to sleep, and patients would be encouraged to get out of bed when they were not trying to sleep. We hypothesized that patients were unaware of the risks of prolonged immobilization and that the lack of an alternative comfortable place for patients to sit other than their bed contributed to their immobility. Previous studies have demonstrated that mobility protocols improve patient-centered outcomes in narrow patient populations such as post-surgical patients or the elderly, but such interventions have not been studied in a heterogeneous patient population [6]. Additionally, previously studied protocols have been labor-intensive from a nursing standpoint, requiring, for example, that staff provide one-on-one supervised ambulation 3–4 times per day [9]. We aimed to determine whether establishing a relatively low-intensity, pragmatic protocol to discourage patients from spending time in bed would result in shorter length of stay by improving mobility. We further aimed to assess the additional impact of providing a recliner chair, a safe and comfortable alternative to their bed on these outcomes.

## Materials and methods

BRAVE (Bed Rest AVoidancE) was a prospective, controlled investigation of a novel intervention intended to reduce time subjects spent in bed. The intervention was conducted at Los Angeles County + University of Southern California (LAC+USC) Medical Center from March 20, 2017 through September 19, 2017. LAC+USC is a public, urban hospital serving predominantly inner-city minority, uninsured, indigent patients. The intervention and control wards were symmetrically laid out. All wards consisted of medical/surgical beds without cardiac telemetry monitoring. To increase generalizability of the study results, subjects were admitted per clinical routine, with bed assignments made by the bed control department, which was not aware of the study.

Handouts (S1 Fig) were developed at a 3^rd^ grade level that informed subjects and their families about the risks of bed rest and encouraged the subjects to only lie in bed when they were attempting to sleep. Handouts were translated into 5 languages (Spanish, Korean, Tagalog, Mandarin, Vietnamese). Nursing staff on the intervention wards provided these handouts to subjects and their families as part of the standard admission process. In addition, laminated reminder signs identical to the handouts were posted on the wall opposite the head of the bed. Posters were placed in the main entrance of the study units.

During study ramp up, nursing staff, physical therapy, and hospitalist attendings were educated regarding the dangers of bed rest. All were asked to counsel subjects admitted to the intervention wards about the dangers of bed rest and encourage subjects not to spend time in bed during daylight hours. This education was not repeated or reinforced again during the study period. Half of the intervention rooms were also provided with recliner chairs (Winco Vero Care Cliner) that were bariatrics rated to 227kg (500lbs), vinyl-covered for facile disinfection, and had 180-degree swing arms and locking casters. The other intervention rooms and the control rooms had standard hospital chairs in addition to the hospital bed.

The study was approved by the University of Southern California Health Sciences Campus Institutional Review Board on November 1^st^, 2016 via expedited review with a waiver of informed consent.

### Study outcomes

The primary outcome measure was the length of hospital stay. Subjects’ age and gender were collected in order to control for these factors in the primary analysis. The Medicare Severity Diagnosis Related Group (MS DRG) relative weight for each patient was collected to control for illness severity.

As a secondary endpoint, functional status was assessed using the “6-Clicks” objective functional scoring system [10–12]. Scores of 1–4 (respectively: unable to perform the task, moderate to maximum assist, minimal assist/supervision, independent) were assigned for six tasks: roll over in bed, supine to sitting, bed to chair, sit to stand, walk in room, and climb 3–5 steps with the aid of railing (total score range 6–24). Scores were measured at several points during each hospitalization, depending on availability of volunteer research assistants, and compared between admissions in intervention vs. control wards. As a process measure, nurses maintained a log of hours per shift that subjects spent in bed. Finally, as a safety measure, the rates of falls per 1000 patient-days were collected.

### Data collection

Data were abstracted using an automated query of the electronic health record’s bed management database (Cerner, Kansas City, MO). All patients who were placed on one of the study wards during the intervention period March 20, 2017 through September 19, 2017—including those admitted from the emergency department, elective medical and surgical admissions, inter-hospital transfers and intra-hospital transfers—were included and followed up through January 16, 2018. Consistent with the intention-to-treat principle, subjects who changed rooms during an admission were classified based on treatment group associated with the first room to which they were assigned. We also tabulated the rate of subject crossover during a single hospital admission; that is, the number of subjects who spent more time in a bed of the group other than that to which they were initially assigned. We also tabulated the fraction of hospital stays that were readmissions of the same subject during the study period. Subject and visit variables (age, sex, hospital room, the presence of a bedrest order, length of stay, MS DRG relative weight) were also abstracted by automated query. Data cleaning was performed in STATA MP 15 (STATACORP, College Station, Texas).

The hours spent in bed were prospectively collected on paper data collection forms by the treating nurse for each admission. The “6-Clicks” functional assessments were conducted by research assistants not otherwise involved in the subjects’ care. Research assistants were instructed to record scores for all subjects, but the dates on which subjects were assessed were selected based on the availability of the volunteer research assistants.

Data on patient falls tabulated at the ward level by month were obtained from hospital administrative records. Falls data included the 12 months prior to the intervention as a baseline.

### Statistical analysis

The subjects were exposed to one of three treatments: a behavioral intervention designed to decrease bedrest, a behavioral intervention combined with the provision of a recliner chair, or standard care. The effects of these three conditions were estimated in a single integrated model, with one term capturing the effect of the behavioral intervention and another term capturing of the effect of the recliner chair.

Assuming a non-parametric (right-skewed) distribution of length of stay, a mean length of stay of 8 days, a standard deviation of 17 days and acceptable type I error rate of 5%, analysis 6076 discharges would be necessary to detect a change of length of stay of 1 day with 90% power. Given a total of 37 intervention beds and 37 control beds, with a 90% utilization rate, we decided to collect data over a 6-month period.

For the primary analysis, a generalized estimating equation was used to test differences in length of stay between the control and intervention groups. Because the distribution of length of stay was right-skewed, these values were log-transformed prior to analysis. The model included the following covariates: an indicator variable for behavioral intervention, an indicator variable for recliner chair, age (as a factor by quintiles), MS DRG relative weight, and an indicator variable for whether a particular patient room was physically capable of accommodating a recliner chair. Because observations on the same ward were likely to be correlated, a clustering factor was added at the level of the bed. Thus, the model simultaneously estimated the separate effects of the behavioral intervention and the presence of the recliner chair.

The indicator variable for capability of a room to accommodate a chair was added to the model after an early post-hoc analysis of the data indicated that there was a substantial imbalance in the MS DRG case mix between intervention rooms with recliner chairs and those without. Upon investigation, it was found that rooms that could physically accommodate chairs were also preferentially used for a substantially different population of patients than those that could not. For example, single-patient rooms designated for patients who require respiratory isolation could accommodate recliner chairs whereas 2-patient rooms that were generally used for lower-acuity patients could not. However, there were rooms on the control ward that were identical to either the recliner-capable rooms or recliner-incapable rooms on the intervention ward. Therefore, an indicator variable for the ability of a room to accommodate a recliner chair was added to the model to capture the effect of case mix differences between recliner-capable and recliner-incapable rooms on both intervention and control wards.

The 6-click functional scores were compared using a linear mixed-effects model with one random effect per subject and fixed effects for the same covariates included in the primary analysis, with the addition of a continuous variable for day of the study.

Three post-hoc robustness analyses were conducted. The first added a random effect for subject room and the second excluded all but the first visit of subjects who had multiple visits during the study period. Based on reviewer comments, we added a third propensity-score weighted robustness analysis to account for non-random allocation of subjects. A propensity score was created using subject age, gender, DRG weight, and the specific medical or surgical service to which the subject was admitted. The propensity score was then used a covariate in the generalized estimating equation with treatment group.

The total daily hours spent in bed, a measure of the effectiveness of the protocol in changing subject behavior, was analyzed with a linear mixed effects model with one random effect per subject and fixed effects for the same covariates as the primary analysis.

The rates of falls between intervention and control wards were compared using data from the pre-intervention and post-intervention period in a Poisson regression, with the assumption that fall events were uncorrelated. Analysis was performed using R (Version 3.2.2 Vienna, Austria).

## Results

**Fig 1:**
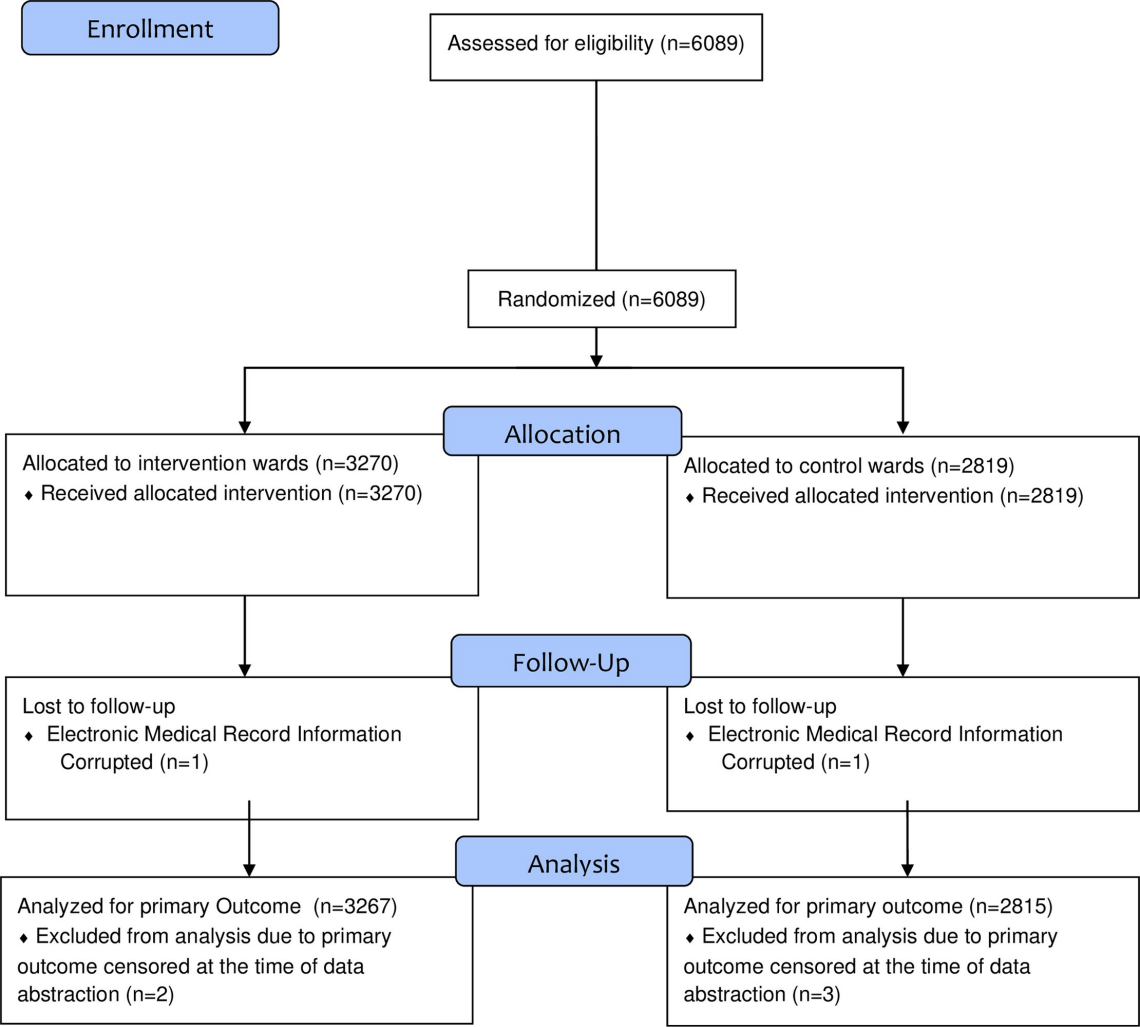
CONSORT diagram.

During 6 months of study, 6089 admissions to the study wards were abstracted from the electronic health record. Of these, a total of 7 admissions were excluded: two admissions for corrupt electronic health record data and five admissions because the patient had not yet been discharged at the time of data abstraction, two weeks after the conclusion of the study period (Fig 1). Thus, a total of 6082 admissions were included in the final analysis. Of these, 672 (11%) represented readmissions of the same subject during the study period. These were included in the final analysis, with each stay analyzed as an independent event. For the secondary outcome of “6-Clicks” functional scores, data on 778 patient stays, including a total of 1725 functional score measurements, were collected by study personnel. Regarding the documentation of hours spent in bed, nurses recorded data on 908 patient stays, including a total of 4268 daily measurements.

The median age, gender distribution and medical service of admission were significantly different between control and intervention groups (Table 1). This imbalance is likely due to the fact that subjects were allocated by bed control as part of routine clinical care. The median MS DRG relative weight as well as the proportion of subjects on bedrest did not differ significantly between intervention and control groups.

**Table 1:** Subject demographics (N = 6082).

	Intervention Rooms	Control Rooms	P value
**Number of Subjects**	3267	2815	
**Clinical Service**			<0.01*
Medicine	2124	2027	
Surgery	800	486	
Neurosurgery	88	62	
Neurology	77	99	
Orthopedic	84	67	
Other	94	74	
**Median Age**	52 (IQR 40–62)	54 (IQR 41–62)	<0.01*
**%Male**	1971 (60.3%)	1825 (64.8%)	<0.01*
**Median MS DRG Relative Weight**	1.8 (IQR 1.1–3.4)	1.8 (IQR 1.1–3.4)	0.84
**%Subjects with Bed Rest Order**	914 (27.9%)	837 (29.5%)	0.14

*Statistically significant

The primary outcome of hospital length of stay in the multivariate model was not affected by either the behavioral intervention or the provision of a recliner chair (Table 2). Median length of stay was 84 hours (IQR 44–175 hours) in the control group, 80 hours (IQR 44–155 hours) in the group who received the behavioral intervention alone, and 88 hours (IQR 44–185 hours) in the group that received both the behavioral intervention and the recliner chair. Only the MS DRG relative weight and top two quintiles of age were associated with a significant difference in the length of stay, with higher values associated with increased length of stay. The results of the three robustness analyses did not yield results that were qualitatively different from the main analysis. A small fraction of admissions, 6%, would have been reclassified if analyzed according to treatment received instead of intention to treat, with the treatment received defined as the study ward in which the subject stayed for the greatest number of hours.

**Table 2:** Multivariate analysis of factors associated with length of stay (N = 6082).

Covariate	% Change in Length of Stay	P value	95% CI
MS DRG Relative Weight^†^	9.2%	<0.01*	8.0–10.4%
Behavioral Intervention	-3.3%	0.39	-10.5–4.5%
Recliner in room	2.0%	0.74	-9.5–14.0%
Room Able to Accommodate Recliner	7.2%	0.12	-1.7–17.0%
First Quintile of Age (≤36 years)	Reference	Reference	Reference
Second Quintile of Age (37–48 years)	-5.8%	0.15	-13.2–2.1%
Third Quintile of Age (49–56 years)	2.2%	0.54	-4.7–9.7%
Fourth Quintile of Age (57–64 years)	12.5%	<0.01*	3.8–21.9%
Fifth Quintile of Age (≥65 years)	11.5%	<0.01*	3.0–20.8%

*Statistically significant

**†**Effect of a difference of one point on the MS DRG relative weight scale (Range 0.5 to– 26.1)

In the multivariate analysis, the secondary outcome of the “6-Clicks” functional score was also not significantly associated with either of the interventions (Table 3). The median score was 17 points in the control group (IQR 10–24 points), 20 points (IQR 14–24 points) in the intervention group without recliner chairs, and 21 points (IQR 12–24 points) in the intervention group with recliner chairs. The MS DRG relative weight and the fifth quintile of age were associated with a significant decrease in the functional score.

**Table 3:** Multivariate analysis of “6-clicks” functional status score (N = 778).

Covariate	Effect on Six Clicks	P value	95% CI
Day of Study	0.0015	0.90	-0.022–0.025
MS DRG Relative Weight	-0.31	<0.01*	-0.44 –-0.19
Behavioral Intervention	0.45	0.44	-0.69–1.61
Recliner Chair	-0.61	0.46	-2.22–1.00
Room Able to Accommodate Recliner	0.12	0.84	-1.03–1.26
First Quintile of Age (≤36 years)	Reference	Reference	Reference
Second Quintile of Age (37–48 years)	0.28	0.67	-1.0–1.6
Third Quintile of Age (49–56 years)	-0.45	0.47	-1.67–0.77
Fourth Quintile of Age (57–64 years)	-0.86	0.17	-2.10–0.48
Fifth Quintile of Age (≥65 years)	-2.2	<0.01*	-3.54 –-0.93
Interaction between Day of Study and Behavioral Intervention	0.019	0.52	-0.039–0.076
Interaction between Day of Study and Recliner Chair	0.0071	0.83	-0.059–0.073

*Statistically significant

Subjects in the control group spent a mean of 21 hours per day in bed (IQR 20–24) versus 20 hours per day in bed (IQR 17–23) in the group with behavioral intervention alone and 19 hours per day (IQR 16–24) in the intervention group who received both the behavioral intervention and the recliner chair. The analysis of bedrest showed a small but statistically significant association between decreased time in bed and the interaction term between study day and the presence of a recliner chair. This reduction in time was quite small: equivalent to 4 fewer minutes in bed per day for each day of study enrollment (i.e., equivalent to 4 minutes less in bed on day 1, 8 minutes less on day 2, etc.) (Table 4). The MS DRG relative weight and the capability of a room to accommodate a recliner were associated with a small increase in the time spent in bed.

**Table 4:** Multivariate analysis of factors associated with time spent in bed (N = 908).

Covariate	Effect on Number of Hours in Bed	P value	95% CI
Day of Study	-0.024	<0.01*	-0.036 –-0.011
MS DRG Relative Weight	0.14	<0.01*	0.06–0.22
Behavioral Intervention	-0.63	0.07	-1.31–0.045
Recliner in room	-0.42	0.40	-1.39–0.53
Room Able to Accommodate Recliner	0.72	0.032*	0.06–1.38
First Quintile of Age (≤36 years)	Reference	Reference	Reference
Second Quintile of Age (37–48 years)	-0.41	0.28	-1.17–0.34
Third Quintile of Age (49–56 years)	0.11	0.77	-0.63–0.84
Fourth Quintile of Age (57–64 years)	0.27	0.47	-1.01–0.47
Fifth Quintile of Age (≥65 years)	0.70	0.07	-0.06–1.46
Interaction between Day of Study and Behavioral Intervention	0.005	0.74	-0.024–0.034
Interaction between Day of Study and Recliner Chair	-0.071	<0.01*	-0.105 –-0.036

*Statistically significant

In the year prior to the intervention, the mean rate of falls per 1000 patient-days in the control wards and intervention wards was 2.1 and 2.5 respectively. In the post-intervention period, the rate was 2.9 in the control wards and 4.1 in the intervention wardsThe increase in the incidence rate ratio for falls between the intervention wards compared to the control wards in the post-intervention period was not statistically significant (S1 Table).

## Discussion

The negative consequences of inadvertent bed rest include loss of muscle mass, decreased ability to complete activities of daily living, increased risk of falls, longer lengths of stay, and greater costs of hospitalization [1–4, 6, 8, 13–15]. Because of the negative impact of bed rest, increasing focus has been applied to early mobilization programs for patients in intensive care units (ICUs).(6) Multiple studies have also demonstrated the efficacy of mobilization protocols in reducing length of stay and increasing the functional status of patient admitted to non-monitored beds.(6) However, it is our experience that such protocols have not been widely adopted in this patient population. A multicenter survey conducted at 3 large medical centers found that the biggest barrier to the implementation of mobility interventions was staff perception of the associated increase in workload(16), suggesting that resource-intensive protocols, such as those that require direct supervision of mobilization, may be impractical to implement.

We sought to determine if a less resource-intensive intervention—consisting of patient and staff education combined with the provision of recliner chairs—applied to a broad, heterogeneous population of inpatients could achieve results similar to those of proven, but more intensive, interventions. Our clinical endpoints were length of stay and 6-clicks functional status score. Unfortunately, we found minimal reduction in time spent in bed in this prospective, controlled study. Likely as a result of this, the intervention did not affect either the length of stay or the “6-clicks” functional status score.

In this study, the analysis of hours in bed suggests a very small effect of the presence of a recliner chair and educational interventions on time spent in bed, approximately 4 minutes per day per number of days enrolled in the study. This finding implies that a more active and intensive intervention may be necessary to encourage mobilization. Indeed, our findings are similar to the 2003 study of by Mundy et al of a similarly low-intensity mobilization protocol for patients with community-acquired pneumonia [16]. In that study, patients were simply encouraged by their nurses to get out of bed once in the first 24 hours of their hospital stay. The decrease in length of stay in the intervention group appeared to just reach statistical significance.

Another possible explanation for our results is that lack of staff and patient education regarding the dangers of bed rest and lack of a comfortable alternative to lying in bed were not the main barriers to patient mobility. In fact, the largest published survey regarding barriers to implementation of early mobility programs for hospital inpatients found that nurses and physical therapists both stated that they regularly educated their patients on the dangers of bedrest and believed that mobilized patients have better outcomes [17]. The study identified perceived increase in workload associated with mobilization as well as low confidence in the ability to safely mobilize inpatients as higher barriers than lack of education about the dangers of bed rest.

### Limitations

Participants in the study were allocated to intervention groups according to the ward they were assigned to as part of usual care, rather than randomized at the level of the individual. Although we tried to account for potential confounders in our analysis, it is possible that there were unmeasured confounders correlated with both the ward assignment and length of stay.

Due to the limited availability of volunteer research assistants the secondary 6-clicks outcome was collected on only 13% of the admissions in the study. Similarly, the outcome of hours in bed was collected for only 15% of admissions. Thus, the conclusions that can be drawn from this data are limited by its high likelihood of bias.

## Conclusion

Our data indicate that providing a passive educational tool, and the provision of a recliner chair were insufficient to reduce the amount of time patients spent in their hospital bed and associated sequelae—loss of functional status and increased in length of hospital stay. Additional research is needed to identify effective, pragmatic strategies to decrease unnecessary bedrest for inpatients outside of the ICU.

## Supporting information

S1 Fig Supplemental figure: Educational patient handout. The handout has been modified from its original version to omit copyrighted images.
(TIF)

S1 Table Effect of intervention on incidence rate ratio of falls per ward. (DOCX)

S1 File Original BRAVE trial protocol. (DOCX)

S2 File TREND statement checklist. (PDF)
