# Supplementary figures and images for: A randomized trial of a behavioral intervention to decrease hospital length of stay by decreasing bedrest
Source: PLoS One. 2020 Jan 10;15(1):e0226332. doi: 10.1371/journal.pone.0226332 (PMC6953761; doi:10.1371/journal.pone.0226332)

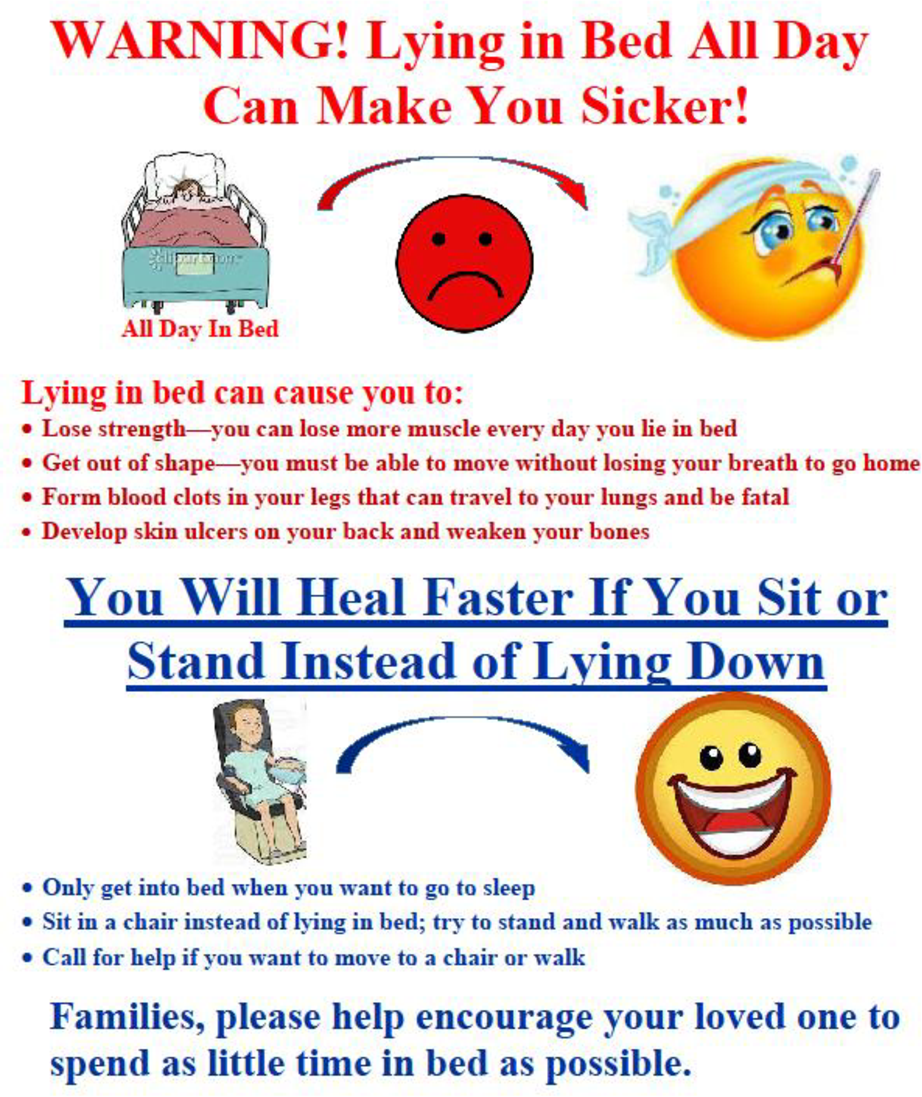

Supplement: S1 Fig — The handout has been modified from its original version to omit copyrighted images. (TIF) [file pone.0226332.s001.tif]
