# Supplementary material for: A randomized trial of a behavioral intervention to decrease hospital length of stay by decreasing bedrest
Source: PLoS One. 2020 Jan 10;15(1):e0226332. doi: 10.1371/journal.pone.0226332 (PMC6953761; doi:10.1371/journal.pone.0226332)
Supplement: S1 Table — (DOCX) [file pone.0226332.s002.docx]

**Supplemental Fig: Educational Patient Handout**

| **Covariate** | **Point Estimate** | **P value** | **95% CI** |
| --- | --- | --- | --- |
| Intervention | 1.2 | 0.58 | 0.6– 2.6 |
| Indicator for Pre/Post Study Initiation Date | 1.4 | 0.39 | 0.7 – 3.0 |
| Interaction term between Pre/Post and Intervention | 1.2 | 0.74 | 0.4 – 3.2 |

**Supplemental Table: Effect of Intervention on Incidence Rate Ratio of Falls per Ward**
