# Supplementary material for: A randomized trial of a behavioral intervention to decrease hospital length of stay by decreasing bedrest
Source: PLoS One. 2020 Jan 10;15(1):e0226332. doi: 10.1371/journal.pone.0226332 (PMC6953761; doi:10.1371/journal.pone.0226332)
Supplement: S1 File — (DOCX) [file pone.0226332.s003.docx]

**II. Organizing Information**

1. Brief History, Mission, and Expertise of the Hospital

LAC+USC Medical Center is a tertiary care, academic public hospital in downtown Los Angeles. The original 100-bed hospital was founded in 1878, replaced by the 2100 bed General Hospital in 1933, and subsequently moved to its current, modern 676 inpatient bed footprint in 2008. The hospital’s mission is to maximize medical care for residents of Los Angeles County, irrespective of ability to pay. The hospital cares for a predominantly indigent (>85% have Medicaid, a similar county-funded program, or are self-pay), minority (>70%), under-privileged, population. It is the lynchpin of the safety net program in LA County, and represents 1/3 of the budget and healthcare resources available to the Department of Health Services in LA County. The hospital provides tertiary/quaternary care across all domains of medicine except for organ transplantation. It provides primary care, specialty outpatient care, and level 1 trauma inpatient care with all medical specialties available.

1. Description of Current Programs, Activities, and Accomplishments

LAC+USC Medical Center handles approximately 31,000 admissions per year, approximately 160,000 patient visits per year in the Emergency Department (ED), making it one of the busiest EDs in the country, and more than 500,000 outpatient visits per year. Its level 1 trauma center handles nearly 40% of the trauma runs in LA County, and by volume handles twice as many trauma runs as the second busiest trauma center in the county (~4000 vs. ~2000 trauma runs). The Medical Center houses one of only seven level 3 NICUs in LA County, and one of only three Burn Units in LA County. It also houses the only hospital jail ward in LA County. It is one of the largest medical training facilities in the US, housing approximately 950 medical residents and fellows, as well as being a primary home for a large nursing college, pharmacy school, and allied health professional training facilities.

LAC+USC is transforming its care delivery to meet the demands of 21^st^ century healthcare in the US. Affordable Care has flipped our care delivery model, and we are focused on becoming a primary care medical home, and delivering high quality, efficient, patient-centered healthcare. Within the last two and a half years, the Medical Center has had a new Chief Executive Officer, Chief Medical Officer, Chief Quality Officer, Associate Medical Director for Quality/Risk/Safety, Associate Medical Director for Inpatient Services, and Associate Medical Director for Regulatory Affairs. We are transforming patient flow in our hospital, and have achieved substantial improvements in ED wait times, in inpatient bed availability, and in patient harm events. We have also implemented a fully integrated electronic medical record. The 4 pillars of our quality program are to: 1) Improve patient satisfaction; 2) Improve publicly reported quality measures; 3) Improve patient flow; and 4) Improve transitions of care. Our completely revamped quality program has dozens of active projects underway across all departments, and incorporating front line staff and resident physicians, to achieve these goals.

1. Demographic Description of the Constituency Served

Based on data from the full year of 2014-2015, LAC+USC serves a very diverse population of patients that includes:

1. Gender: 50% male and 50% female
2. Age: the full spectrum from 0 to greater than 100 years of age, including 16 patients > 100 years, with a median age in the 40-49 range
3. Ethnicity: 55% of patients are Hispanic, with sizable populations of African American, Asian, and Pacific Islanders as well
4. Service area: the hospital serves patients from across the entire county, with 31% from the Metro area, 17% from East/Antelope Valley, 25% from San Gabriel/San Fernando Valley, 20% from South LA, and 2.5% from the South Bay and 2% from the West region
5. Zip Codes: the hospital served patients from more than 1250 zip codes, including all of the UniHealth required zip codes.
6. Hospital Financials
7. Operating Margin: the hospital does not have an operating margin per se, because it is a non-profit, publicly funded organization
8. Days cash on hand: this term cannot be calculated as we are publicly funded and allocations of funding are based on budgets set by the Board of Supervisors—since we are taxpayer funded, the resources available are limited by discretionary spending by government officials.
9. Debt to capital ratio: as above, this term is not meaningful for a publicly funded hospital
10. Payor mix by category: 12% Medicare, 80% MediCal, 4% private, 4% other

IV. Measurable Objectives, Key Activities, and Evaluation Indicators

1. What do you ultimately hope to accomplish through your program/project?

We hypothesize that establishing an innovative protocol to discourage patients from lying in bed unless they are sleeping will shorten lengths of stay by preventing the deconditioning that results from prolonged bed rest. We further hypothesize that the effect of the protocol may be magnified by providing patients with a safe and comfortable reclining chair as an attractive alternative to lying in bed. **In essence, the overarching goal of our intervention is to fundamentally flip the culture of inpatient medicine such that beds are viewed as places to sleep, and patients are expected not to be in bed unless they are trying to sleep.**

A common cause of prolonged length of stay in the hospital is the rapid deconditioning that occurs after patients are admitted.^1-5^ Up to 50% of inpatients suffer functional decline due to bed rest after admission, and even in-patients who are not placed on bed rest by their physicians spend an astonishing 20 out of 24 hours lying in bed.^2,4-7^ The negative consequences of this inadvertent bed rest include loss of muscle mass, decreased ability to complete activities of daily living, increased risk of falls, longer lengths of stay, and greater costs of hospitalization.^1,2,4,6-11^ Because of the negative impact of bed rest, increasing focus has been applied to early mobilization programs for patients in intensive care units (ICUs).^1^ However, only 10% of admissions are to ICUs, and there have been few efforts to similarly mitigate the impact of bed rest on patients admitted to non-monitored rooms.

One of the primary reasons patients spend so much time in bed is that the culture of medicine in most hospitals is simply to assume that patients will spend the majority of their time lying in bed. As a result, the patients become deconditioned. A related problem may be that chairs in patients’ rooms may be difficult to move/maneuver for debilitated patients who have difficulty rising to their feet from a low, seated position.

**We seek to disrupt the normal practice of inpatient medicine by educating patients and their families that hospital beds are for sleeping only, and that patients should spend their awake hours out of their beds.**

1. What are the measurable objectives of your program/project?

We will conduct an active-controlled investigation in which the bed-rest reduction protocol is implemented either with or without special reclining chairs, compared to control rooms with no intervention. We will compare length of hospital stay, time spent in bed, functional status using a validated instrument, falls, pressure ulcers, deep vein thrombosis, and hospital acquired pneumonia for patients admitted to interventional vs. control rooms. To ensure equivalent patient populations, we will also compare and control for any differences in case mix index, severity of illness, and risk of mortality. The primary analysis will be intention-to-treat combining intervention rooms with and without chairs compared to control rooms. Prioritized secondary analyses will compare intervention rooms with vs. without chairs, and intervention vs. control rooms in the per protocol population (excluding patients on bed rest or with medical conditions precluding leaving the bed).

1. What are the key activities of your program project?

The bed rest-avoidance intervention will have both active and passive components.

1. Passive Intervention

Patient handouts (see below) have been developed that inform patients and their families about the risks of bed rest, and encourage the patients to only lie in bed when they are attempting to go to sleep. These handouts are written at a 3^rd^ grade level to facilitate understanding, and have been vetted by our communications team which is accustomed to preparing written materials for our safety net, indigent population. Nursing staff on the interventional ward will provide these handouts to patients and their families as part of the standard admission intake process. We will use grant funds to pay for professional translation into 5 languages (Spanish, Korean, Tagalog, Mandarin, Vietnamese) which reflect the most frequent non-English speaking patients we encounter.

In addition, laminated reminder signs identical to the handouts will be posted on the wall opposite the head of the bed.

**Educational Handout for Patients and Families and to be Posted Opposite the Bed**

**WARNING! Lying in Bed All Day**

**Can Make You Sicker!**

**Lying in bed can cause you to:**

- **Lose strength—you can lose more muscle every day you lie in bed**
- **Get out of shape—you must be able to move without losing your breath to go home**
- **Form blood clots in your legs that can travel to your lungs and be fatal**
- **Develop skin ulcers on your back and weaken your bones**

**You Will Heal Faster If You Sit or Stand Instead of Lying Down**

- **Only get into bed when you want to go to sleep**
- **Sit in a chair instead of lying in bed; try to stand and walk as much as possible**
- **Call for help if you want to move to a chair or walk**

**Families, please help encourage your loved one to spend as little time in bed as possible.**

1. Active Intervention

During study ramp up, nursing staff, physical therapy, and hospitalist attendings will be in-serviced to the dangers of bed rest. This multi-disciplinary team will be asked to counsel admissions to intervention wards about the dangers of bed rest and encourage patients not to spend time in bed during daylight hours. The team will also encourage patients to do self-directed functional exercises with physical therapy guidance.

Physical therapy will provide routine assessment and care when consulted for patients in the interventional rooms, with the only difference being the availability of the recliner chairs. Consultation of physical therapy will not be mandatory, and will be ordered only when the primary team deems it necessary per clinical routine (i.e., consultation is not affected by the study).

1. Reclining Chair

The investigators, working with operations, nursing, physical therapy, and environmental services leadership, have evaluated potential chairs to meet the needs of the study. We screened numerous potential recliner chairs to meet very specific criteria required for use in general ward rooms. Specific required criteria included: 1) easy to clean surface, excluding fabric covering, for infection control and environmental services cleaning purposes; 2) rated to ≥ 400 lbs of body weight; 3) adjustable foot rest and reclining for comfort, and reclinable fully flat or to trendelenberg for emergencies; 4) easily mobile so the chair can be moved in the patient room, but with wheel locks; and 5) adjustable arms for ease of patient transfer and managing lines. Of the numerous chairs evaluated, we found several that met various of these criteria and solicited competitive quotes. The Winco Extra Large Care Cliner was ultimately selected for testing; it has a 450 pound weight limit, lockable wheels, infection control-compliant covering material, even distribution of body weight on padded cushions with ergonomic headrest, “one touch” droppable side arms, infinitely adjustable back including to trendelenberg, and costs < $1600 per chair.

1. What evidence supports the efficacy of the proposed intervention?

Deconditioning of hospital inpatients has been well described to lead to a host of new medical problems, including delirium, sensory deprivation, depression, isolation, skin breakdown, loss of muscle tone, and loss of bone density.^1,4,8,9^ But the most immediate and obvious impacts are loss of lean muscle mass (up to 5% per day), reduced exercise capacity, and loss of ability to complete activities of daily living (ADLs).^1,2,4,6,7^ This process has been referred to as deconditioning, or “functional decline.” In turn, functional decline results in prolongation of hospital stays, requiring additional hospital resources to rehabilitate the patient so that it is safe to transition them to home or the next venue in the care continuum.^1,2,6,7^

Up to 50% of hospital inpatients suffer consequences from deconditioning, including prolongation of hospitalization, adverse events including falls and pressure ulcers, and even excess mortality and substantial increases in hospital costs.^2,4,6,7^ The loss of functional ability and exercise tolerance happens remarkably fast, as early as hospital day 2 (24-48 hours).^1,3,6,7,10^ Furthermore, loss of functional ability due to deconditioning led to a >20% increase in DRG-adjusted hospital costs in one study, an impact that was maintained after multivariate analysis to control for confounders.^11^

Multiple studies have explored the potential for early mobilization programs to reduce the impact of deconditioning in intensive care units (ICUs), status post stroke, and after several specific types of surgeries.^12-21^ Collectively, these niche patient populations account for < 10-15% of all hospital admissions. However, a recent systematic review of the literature identified only 9 studies specifically evaluating the potential for early mobilization programs to more generally impact the 85-90% of patients admitted to medical-surgical ward beds.^1^ These studies were specific to patients with deep venous thrombosis, community acquired pneumonia, or several types of surgeries. In general such programs resulted in shorter recovery times post-operatively, and several found reductions in hospital lengths of stay of 1 day or more. The most effective programs were those with well-described, standard protocols. However such protocols tended to involve prescription of specific exercises for patients while in bed, and encouraged excursions from bed several times per day.

A more fundamental program, designed to keep patients from lying in bed in the first place, has not been described. Patients who are hospitalized spend a very large percentage of their time (>85%) lying in bed; this is true even of patients who were ambulatory prior to hospitalization.^5^

We seek to describe a bed-rest prevention protocol that is applicable to all patients admitted to ward beds, based on active and passive interventions combined with use of a safe, comfortable, and functional recliner chair. If we can fundamentally change the use of beds by inpatients, such that they are only used while sleeping at night, and patients spend their days out of their beds, the potential is to dramatically reduce the incidence of significant bed-rest induced deconditioning, with attendant improvements in patient outcomes.

1. What are your evaluation indicators and benchmarks for success? How will you determine if your project has accomplished its ultimate objective?

Our primary outcome measure is average length of stay, which will be compared for patients admitted and discharged during the 6 month study in rooms with the intervention vs. concurrent control rooms in which there is no intervention. We will compare length of stay in rooms with vs. without the reclining chair (secondary analysis). Length of stay will be obtained retrospectively after the study period by query, stratified by ward, and room number within the ward (to distinguish rooms that had recliners vs. not). The primary analysis will be conducted in the intention-to-treat (ITT) population, defined as all patients admitted to both wards. A prioritized secondary analysis will be conducted in the per protocol (PP) population, defined to exclude patients for whom a bed rest order was placed or those with medical conditions precluding getting out of bed (e.g., patients on mechanical ventilation, those with advanced dementia who are chronically bed bound).

Nurses will keep logs of hours per shift patients spent in bed vs. out of bed. These logs will have a medical record number at the top of the sheet to enable capture of times spent in bed per patient. However, during manual data entry into REDCap, each paper form will also have a unique, sequential study ID number on it, coded as ward location (A or B), reclining chair present or not, and admission number (see Appendix B for draft case report form). Medical record number will be specified as a personal identifier on the REDCap database, required for linkage to hospital data; only the study coordinator will have access to this field, and data exports will be restricted to exclude the medical record number. Paper logs will be maintained in a locked cabinet in the PI’s office until completion of statistical analysis, at which time they will be shredded. Study coordinators will be responsible for ensuring that the logs are maintained on a daily basis by reminding nursing staff when logs have not been completed. Coordinator coverage will be funded for 7 days per week. Coordinators will enter the data into the electronic REDCap database.

For the functional assessment, study coordinators will apply the well-validated “6-Clicks” objective scoring system for patients. In brief, scores of 1-4 (unable, moderate to maximum assist, minimal assist/supervision, independent) are applied across 6 domains: roll over in bed, supine to sitting, bed to chair, sit to stand, walk in room, 3-5 steps with a rail (total score range 6-24). Scores will be entered on case report forms once per day. We will analyze scores both cumulatively (across all domains) and within each separate domain. Analysis will include both change in score from admission to discharge, and final score at discharge, compared for patients in the intervention vs. control rooms. Falls and pressure ulcers are already captured prospectively as part of routine hospital monitoring; we will compare fall data on intervention wards vs. control wards both because decreasing deconditioning could decrease the risk of falls, but also because patients spending more time out of bed could cause increased falls. Thus falls are both an efficacy measure and a primary safety measure to ensure that the intervention is not creating excess risk for patients. The number of falls and pressure ulcers per month are reported as aggregate data with no patient identifiers.

Given 32 intervention beds, with a 90% utilization, an average baseline length of stay of 4.8 (+/- 3.5) days, and 180 days (6 months) of study duration, 1080 discharges are expected on each of the intervention and control wards (2160 total discharges). We anticipate some Hawthorne effect impact in the control rooms, given the fact that physicians and physical therapists will have patients on both the control and intervention wards. Thus, to ensure adequate power, we conservatively assume the control room average length of stay will decrease to 4.5 days. We hypothesize that the intervention will achieve a further 0.5 day average reduction in length of stay. Given these parameters, the study will have a 90% power to detect an average reduction of 0.5 day length of stay, assuming an underlying standard deviation to length of stay of 3.5 days as is true of the baseline data (two-sided α = 0.05). Alternatively there will be >95% power to compare two Poisson-distributed means of 4.5 vs. 4.0. For the prioritized secondary analysis of length of stay change in intervention rooms with vs. without reclining chairs (n=540 per group), the study will have an 80% power to detect a difference of 0.6 days assuming a 3.5 day standard deviation in the intervention rooms.

For time spent in bed and 6-click scores, conservatively assuming 25% of the data are missing, the study will have a 90% power to detect an average 2 hour decline in time spent in bed (18 hours vs. 20 hours) assuming a 12 hour standard deviation in times spent in bed, and a 2 point difference in 6-click score assuming a 12-point standard deviation (scores from 6-24).

**References**

1. Pashikanti L, Von Ah D. Impact of early mobilization protocol on the medical-surgical inpatient population: an integrated review of literature. Clinical nurse specialist CNS 2012;26:87-94.

2. Brown CJ, Friedkin RJ, Inouye SK. Prevalence and outcomes of low mobility in hospitalized older patients. Journal of the American Geriatrics Society 2004;52:1263-70.

3. Hirsch CH, Sommers L, Olsen A, Mullen L, Winograd CH. The natural history of functional morbidity in hospitalized older patients. Journal of the American Geriatrics Society 1990;38:1296-303.

4. Suesada MM, Martins MA, Carvalho CR. Effect of short-term hospitalization on functional capacity in patients not restricted to bed. American journal of physical medicine & rehabilitation / Association of Academic Physiatrists 2007;86:455-62.

5. Brown CJ, Redden DT, Flood KL, Allman RM. The underrecognized epidemic of low mobility during hospitalization of older adults. Journal of the American Geriatrics Society 2009;57:1660-5.

6. Graf C. Functional decline in hospitalized older adults. The American journal of nursing 2006;106:58-67, quiz -8.

7. King BD. Functional decline in hospitalized elders. Medsurg nursing : official journal of the Academy of Medical-Surgical Nurses 2006;15:265-71; quiz 72.

8. Convertino VA. Cardiovascular consequences of bed rest: effect on maximal oxygen uptake. Medicine and science in sports and exercise 1997;29:191-6.

9. Creditor MC. Hazards of hospitalization of the elderly. Annals of internal medicine 1993;118:219-23.

10. Sands LP, Yaffe K, Covinsky K, et al. Cognitive screening predicts magnitude of functional recovery from admission to 3 months after discharge in hospitalized elders. The journals of gerontology Series A, Biological sciences and medical sciences 2003;58:37-45.

11. Chuang KH, Covinsky KE, Sands LP, Fortinsky RH, Palmer RM, Landefeld CS. Diagnosis-related group-adjusted hospital costs are higher in older medical patients with lower functional status. Journal of the American Geriatrics Society 2003;51:1729-34.

12. Bailey P, Thomsen GE, Spuhler VJ, et al. Early activity is feasible and safe in respiratory failure patients. Critical care medicine 2007;35:139-45.

13. Tucker D, Molsberger SC, Clark A. Walking for wellness: a collaborative program to maintain mobility in hospitalized older adults. Geriatric nursing 2004;25:242-5.

14. Baird G, Maxson P, Wrobleski D, Luna BS. Fast-track colorectal surgery program reduces hospital length of stay. Clinical nurse specialist CNS 2010;24:202-8.

15. Cumming TB, Collier J, Thrift AG, Bernhardt J. The effect of very early mobilisation after stroke on psychological well-being. Journal of rehabilitation medicine 2008;40:609-14.

16. Cumming TB, Thrift AG, Collier JM, et al. Very early mobilization after stroke fast-tracks return to walking: further results from the phase II AVERT randomized controlled trial. Stroke; a journal of cerebral circulation 2011;42:153-8.

17. Lee TG, Kang SB, Kim DW, Hong S, Heo SC, Park KJ. Comparison of early mobilization and diet rehabilitation program with conventional care after laparoscopic colon surgery: a prospective randomized controlled trial. Diseases of the colon and rectum 2011;54:21-8.

18. Morris PE. Moving our critically ill patients: mobility barriers and benefits. Critical care clinics 2007;23:1-20.

19. Morris PE, Herridge MS. Early intensive care unit mobility: future directions. Critical care clinics 2007;23:97-110.

20. van Wijk R, Cumming T, Churilov L, Donnan G, Bernhardt J. An early mobilization protocol successfully delivers more and earlier therapy to acute stroke patients: further results from phase II of AVERT. Neurorehabilitation and neural repair 2012;26:20-6.

21. Dammeyer JA, Baldwin N, Packard D, et al. Mobilizing outcomes: implementation of a nurse-led multidisciplinary mobility program. Critical care nursing quarterly 2013;36:109-19.
